# Antimicrobial resistance of *Staphylococcus* spp. isolated from canine specimens submitted to a veterinary diagnostic laboratory in South Africa

**DOI:** 10.14202/vetworld.2025.1421-1432

**Published:** 2025-06-06

**Authors:** Themba Titus Sigudu, James W. Oguttu, Daniel N. Qekwana

**Affiliations:** 1 Department of Agriculture and Animal Health, College of Agriculture and Environmental Sciences, University of South Africa, Johannesburg, South Africa; 2 Division of Health and Society, School of Public Health, Faculty of Health Sciences, University of Witwatersrand, Johannesburg, South Africa; 3 Section Veterinary Public Health, Department of Paraclinical Sciences, Faculty of Veterinary Science, University of Pretoria, Pretoria, South Africa

**Keywords:** antimicrobial resistance, canine infections, multidrug resistance, South Africa, *Staphylococcus*, veterinary surveillance

## Abstract

**Background and Aim:**

The rising burden of antimicrobial resistance (AMR) in veterinary medicine poses significant threats to animal and public health. In South Africa, inadequate surveillance exacerbates the challenge, particularly regarding *Staphylococcus* spp. infections in companion animals. This study aimed to investigate the patterns and predictors of AMR and multidrug resistance (MDR) in *Staphylococcus* isolated from dogs between 2012 and 2017.

**Materials and Methods:**

A retrospective cross-sectional study was conducted on 1627 *Staphylococcus* isolates. Data regarding animal demographics and antimicrobial susceptibility were extracted, cleaned, and analyzed. Intermediate susceptibility results were classified as resistant. AMR was defined as resistance to at least one antimicrobial from one class and MDR as resistance to antimicrobials from three or more classes. Descriptive statistics, Cochran–Armitage trend analysis, and binary logistic regression models were employed to assess trends and predictors of AMR and MDR.

**Results:**

Overall, 61.2% of isolates exhibited resistance to at least one antimicrobial, and 39.0% were classified as MDR. The highest resistance was observed against penicillins (39.64%), followed by aminoglycosides (22.31%). Significant predictors of AMR included *Staphylococcus* species, specimen type, and year of isolation, while MDR was significantly associated with specimen type and the age of the dog. Notably, *Staphylococcus pseudintermedius* showed a markedly higher likelihood of resistance (Adjusted Odds Ratio = 2.23, p < 0.001) compared to other species. Temporal trends indicated a decrease in AMR but an increase in MDR across the study period.

**Conclusion:**

The high prevalence of AMR and MDR among canine *Staphylococcus* isolates, particularly in skin infections and among younger dogs, underscores the urgent need to strengthen antimicrobial stewardship, enhance surveillance systems, and target interventions in veterinary practice. These findings serve as critical baseline data for future assessments of AMR trends and can be used to inform strategies to mitigate the dissemination of resistant pathogens between animals and humans.

## INTRODUCTION

*Staphylococcus* spp. are facultative anaerobic, Gram-positive, circular-shaped bacteria that occur in clusters [1]. They are catalase-producing, non-motile, glucose-fermenting, and non-sporing bacteria. *Staphylococcus* spp. are commonly isolated from the skin and mucosa of dogs as commensal organisms. However, they can also cause diseases such as pyoderma and otitis externa in dogs [2]. Infection usually occurs when the skin or mucosal barriers are compromised by predisposing factors, such as atopic dermatitis, medical and surgical procedures, or immunosuppressive disorders [3]. There are 37 known *Staphylococcus* species, which can be divided into coagulase-positive and coagulase-negative species based on their ability to produce coagulase enzymes that cause blood to clot [4].

Numerous coagulase-negative *Staphylococcus* species (CoNS), such as *Staphylococcus epidermidis*, *Staphylococcus haemolyticus*, *Staphylococcus warneri*, *Staphylococcus lugdunensis*, and *Staphylococcus chromogenes*, are commonly isolated from dogs. These species are generally considered less pathogenic and are often regarded as non-pathogenic commensals of the skin and mucous membranes. However, certain CoNS species, although generally regarded as non-pathogenic, can act as opportunistic pathogens under specific conditions, particularly in immunocompromised individuals or those with underlying health issues. For example, *S. epidermidis* is often associated with infections in patients with implanted medical devices, leading to device-related infections such as catheter-associated bloodstream infections and prosthetic joint infections. *S. lugdunensis*, while typically harmless, has been linked to severe infections, such as endocarditis, osteomyelitis, and soft tissue infections, particularly in individuals with prosthetic devices or compromised immune systems. *S. haemolyticus* is another CoNS species that can cause urinary tract infections, bloodstream infections, and wound infections, especially in vulnerable patients or hospital settings. Similarly, *S. warneri*, usually a skin commensal, has been reported in cases of catheter-related bloodstream infections in immunocompromised individuals. Finally, *S. chromogenes*, although rarely pathogenic, has been implicated in infections such as endocarditis, particularly in patients with prosthetic heart valves. These examples highlight the potential for CoNS species to cause severe infections when the host’s immune defenses are compromised.

On the other hand, CoPS species, particularly *Staphylococcus pseudintermedius*, are considered the primary staphylococcal pathogens in dogs. *S. pseudintermedius* is the most common cause of skin infections, including pyoderma, as well as ear, wound, and urinary tract infections in dogs. It is a major concern in veterinary medicine due to its ability to cause significant morbidity in affected animals and its potential for developing resistance to antibiotics, including methicillin-resistant *S. pseudintermedius* (MRSP), which further complicates treatment. CoPS species are more virulent than CoNS and can cause serious, often acute, infections that require prompt veterinary intervention. Other CoPS species associated with dogs include *S. intermedius*, *S. schleiferi* subspp. *coagulans*, *S. hyicus*, *S. lutrae*, and *S. delphini* [5].

*Staphylococcus* infection in dogs has been identified as a growing concern in animal medicine [6]. They play a significant role in skin and surgical site infections [7] and lead to significant treatment challenges [8]. Moreover, dogs represent a potential source of methicillin-resistant *Staphylococcus aureus* infections and re-infections in humans [9]. Available evidence indicates potential zoonotic transmission of *Staphylococcus* infections between dogs and humans, with companion animals contributing to the spread of resistant strains [10, 11]. Resistance among *Staphylococcus* of dog origin is a significant animal health and public health concern due to the close companionship between dogs and humans. The seriousness of the problem is appreciated when consideration is given to the fact that there are nine million pet dogs within the boundaries of South Africa [12].

Resistance to commonly used antimicrobials, particularly acquired multidrug resistance (MDR) among CoPS species, is a growing concern in both human and veterinary medicine [10]. Antibiotics commonly associated with MDR include penicillins (e.g., ampicillin, amoxicillin), cephalosporins (e.g., ceftriaxone, cefepime), and fluoroquinolones (e.g., ciprofloxacin, levofloxacin). Macrolides such as azithromycin and clarithromycin, as well as tetracyclines like doxycycline, are also frequently implicated in resistance. Aminoglycosides, including gentamicin and tobramycin, along with carbapenems such as meropenem and imipenem, are other examples of antibiotics facing resistance issues. In addition, sulfonamides, such as trimethoprim-sulfamethoxazole, contribute to the problem. These antibiotics are often involved in multidrug-resistant infections, with bacteria employing mechanisms such as efflux pumps, enzyme production (e.g., beta-lactamases), or alterations in target sites to evade treatment effects. Methicillin-resistant *Staphylococci* are important pathogens and are often multidrug resistant, thus extremely restricting treatment options.

Despite significant advancements in antimicro-bials over the last century [13], the increased use of these drugs in humans and domestic animals has led to a rise in antimicrobial resistance (AMR), which has become a global public health issue [14]. This resistance has been increasingly reported in domestic species, especially in canine healthcare.

Despite the growing recognition of AMR among *Staphylococcus* species in companion animals globally, there remains a substantial lack of comprehensive surveillance data in South Africa, particularly concerning canine isolates. Most studies to date have focused predominantly on human-associated *S. aureus* infections, with relatively limited attention given to veterinary-specific pathogens such as *S. pseudintermedius* and other coagulase-positive *Staphylococci* (CoPS) and CoNS isolated from dogs. Furthermore, while emer- ging reports have documented the presence of methicillin-resistant strains and MDR among canine isolates in other regions, there is insufficient epidemiological information detailing the patterns, trends, and predictors of AMR and MDR in South African veterinary settings. In addition, the potential zoonotic implications posed by antimicrobial-resistant *Staphylococcus* strains circulating within the substantial domestic dog population remain poorly understood. The absence of structured, longitudinal data from diagnostic laboratory records further impedes the formulation of effective antimicrobial stewardship programs tailored to veterinary practice in the region. Therefore, there is an urgent need for localized, systematic investigations to elucidate resistance profiles; identify risk factors associated with AMR and MDR; and establish baseline data to support future surveillance, policy development, and intervention strategies.

The primary aim of this study was to investigate the patterns and predictors of AMR and MDR among *Staphylococcus* species isolated from canine clinical specimens submitted to a veterinary diagnostic laboratory in South Africa between 2012 and 2017. Specifically, this study sought to (i) determine the prevalence of AMR and MDR among *Staphylococcus* spp. isolates; (ii) characterize resistance profiles across different antimicrobial classes; (iii) analyze temporal trends in AMR and MDR over the 6-year study period; and (iv) identify demographic and clinical factors, including age, specimen type, and bacterial species, that are associated with an increased likelihood of AMR. By addressing these objectives, the study contributes to generation of foundational epidemiological data necessary for informing targeted antimicrobial stewardship initiatives, improving therapeutic strategies in companion animal medicine, and mitigating the broader public health risks associated with zoonotic transmission of resistant staphylococcal pathogens.

## MATERIALS AND METHODS

### Ethical approval

Access to the veterinary database, including animal and owner information, was restricted to laboratory staff and could only be accessed on the laboratory premises. Accordingly, data extraction was performed by a laboratory staff member, and de-identified data were subsequently provided to the researcher. Confidentiality and anonymity were strictly maintained by ensuring that no patient animal information was included in any articles or reports. In addition, permission to utilize the data was obtained from the veterinary diagnostic laboratory. Ethical approval for the study was granted by the University of South Africa (UNISA) College of Agriculture and Environmental Sciences Health Research and Animal Research Ethics Committees (Reference: 2018/CAES/107). Data were securely protected against unauthorized access, accidental loss, or destruction, by storing all soft copies as encrypted files on computers and flash drives.

### Study period and location

The records were analyzed between 10 and 14 June 2019 at the Veterinary Diagnostic Laboratory in Johannesburg.

### Informed consent

This retrospective study analyzed de-identified secondary laboratory data on *Staphylococcus* species isolates. No additional procedures or interventions were conducted on the dogs or their owners. All laboratory information was kept confidential and accessible only to authorized members of the research team, and no personal identifying information was included in the analysis or dissemination of results.

### Study design and data sources

A retrospective cross-sectional study design was employed to achieve the objectives of the study. Records of 1,627 clinical isolates obtained from dog specimens submitted to a veterinary diagnostic laboratory between January 2012 and December 2017 were analyzed. The records contained both animal demographic information and antimicrobial sensitivity test results.

### Data management

Before analysis, the dataset was thoroughly examined for inconsistencies, including missing information, incorrect addresses, and duplicate entries. No duplicate or mixed infections were identified. For each isolate, the following variables were extracted: Age, sex, species of organism, specimen type, and year and season of submission. The variable “age” was recategorized into five groups: <2, 2–4, 5–6, 7–8, and >8 years. Similarly, months were grouped into four seasonal categories: Autumn (March, April, and May), winter (June, July, and August), spring (September, October, and November), and summer (December, January, and February).

The resistance status variable was reclassified into a binary outcome, with isolates categorized as either resistant (0) or susceptible (1). This was achieved by recategorizing isolates with “intermediate” susceptibility as resistant, based on guidelines provided by the Clinical and Laboratory Standards Institute and the European Committee on Antimicrobial Susceptibility Testing. This approach aligns with standard public health and epidemiological practices, where intermediate results are grouped with resistant isolates to yield a more conservative estimate of resistance. The rationale for this reclassification is based on the potential for clinical treatment failure associated with the intermediate isolates under standard dosing regimens, particularly in immunocompromised patients or infections at anatomical sites with suboptimal drug penetration.

AMR was defined as resistance to at least one antimicrobial class, while MDR was defined as resistance to three or more antimicrobials from three or more antimicrobial drug classes [11].

### Statistical analysis

All data processing and statistical analyses were conducted using Stata Statistical Software, version 17 (StataCorp, 2021, TX, USA). Crude and factor-specific proportions for categorical variables, along with their corresponding 95% confidence intervals (CIs), were calculated based on time, animal demographics, and specimen origin, and results were presented in tabular format. Annual changes in the proportions of *Staphylococcus* spp. were visualized using temporal graphs. The Cochran–Armitage trend test was applied to evaluate temporal trends in resistance among isolates.

To identify predictors of AMR and MDR, univariate and multivariate binary logistic regression models were constructed. In the first stage, univariate models were fitted with “AMR” or “MDR” as the outcome variables and each explanatory variable independently. Variables that demonstrated significance at a liberal threshold of p ≤ 0.2 in the univariate analysis were subsequently included in the multivariate model.

During the second stage, a manual backward selection technique was utilized to refine the multivariate model. Potential confounders were identified by assessing changes in parameter estimates when each variable was removed from the model; a change of ≥20% in any parameter estimate was considered to indicate confounding, warranting the retention of the variable in question in the final model. For each variable retained in the final model, adjusted odds ratios (AORs) and their 95% CIs were computed.

## RESULTS

### Distribution of AMR status based on age and sex of the dog and *Staphylococcus* species

A total of 1,627 *Staphylococcus* spp. isolates were included in this study. Of these, 61.20% exhibited AMR, and 39.00% were classified MDR (Table 1). A slightly higher proportion of AMR isolates originated from male dogs (52.46%) compared with female dogs (47.54%). Similarly, a higher proportion of MDR isolates were derived from male dogs (57.73%) than from female dogs (42.27%) (Table 1).

**Table 1:** Distribution and antimicrobial resistance of canine *Staphylococcus* isolates by age, sex, and species of organism in South Africa, 2012-2017.

Variable	Total isolates			AMR^b^ isolates			MDR^c^ isolates		
		
	*n*	%	95% CI^a^	*n*	%	95% CI^a^	*n*	%	95% CI^a^
Sex	1627			995			634		
Male	866	53.23	0.509–0.556	522	52.46	0.493–0.556	366	57.73	0.539–0.613
Female	761	46.77	0.443–0.492	473	47.54	0.444–0.507	268	42.27	0.384–0.461
Age groups (years)	1627			995			634		
≤2	160	9.83	0.085–0.114	110	11.06	0.091–0.132	81	12.78	0.093–0.162
3–4	440	27.04	0.249–0.293	269	27.04	0.241–0.297	202	31.86	0.278–0.357
5–6	341	20.96	0.191–0.231	201	20.20	0.177–0.227	139	21.92	0.184–0.257
7–8	309	18.99	0.172–0.210	172	17.29	0.150–0.196	111	17.51	0.139–0.210
>8	377	23.17	0.212–0.253	243	24.42	0.218–0.270	101	15.93	0.127–0.193
Organism	1627			995			634		
CoPS^d^	1493			913			587		
*Staphylococcus pseudintermedius*	1392	93.24	0.926–0.938	760	83.24	0.933–0.941	571	97.27	0.955–0.990
*Staphylococcus aureus*	95	6.36	0.059–0.068	56	6.13	0.057–0.064	15	2.56	0.016–0.035
*Staphylococcus intermedius*	6	0.40	0.002–0.006	23	2.52	0.003–0.005	1	0.17	0.001–0.004
CoNS^e^	94			57			25		
*Staphylococcus epidermidis*	83	88.30	0.832–0.934	50	87.72	0.796–0.958	18	72.00	0.688–0.949
*Staphylococcus saprophyticus*	4	4.26	0.009–0.076	3	5.26	0.016–0.089	1	4.00	0.036–0.127
*Staphylococcus chromogenes*	3	3.19	0.004–0.060	2	3.51	0.002–0.067	1	4.00	0.036–0.127
*Staphylococcus lentus*	2	2.13	0.001–0.042	1	1.75	0.015–0.050	1	4.00	0.036–0.127
*Staphylococcus felis*	2	2.13	0.001–0.042	1	1.75	0.015–0.050	1	4.00	0.036–0.127
CoPS/CoNS^f^	40			25			22		
Unspecified *Staphylococcus*	30	75.00	0.616–0.884	24	96.00	0.922–0.998	17	77.27	0.613–0.933
*Staphylococcus schleiferi*	10	25.00	0.116–0.384	1	4.00	0.000–0.080	5	22.73	0.067–0.388

a 95% CI=95% Confidence interval,

b AMR=Antimicrobial resistance,

c MDR=Multidrug resistant,

d CoPS=Coagulase-positive Staphylococci,

e CoNS=Coagulase–negative Staphylococci,

f CoPS/CoNS=Coagulase–Variable Staphylococci

With respect to age, the majority of AMR isolates were obtained from dogs aged 2–4 years (27.04%), followed by dogs aged over 8 years (24.42%). Dogs under 2 years of age contributed the lowest proportion of AMR isolates (11.06%) (Table 1). A similar pattern was observed for MDR isolates, where dogs aged 2–4 years contributed the highest proportion (31.86%), followed by dogs aged 5–6 years (21.92%). Dogs under 2 years of age contributed the lowest proportion (12.78%) of MDR isolates (Table 1).

Based on *Staphylococcus* species, the majority of AMR isolates were CoPS (91.76%). Within this group, *S. pseudintermedius* accounted for the highest proportions of AMR (83.24%) and MDR (97.20%) isolates. *S. aureus* comprised 6.13% and 2.56% of AMR and MDR isolates, respec-tively, while 2.52% of AMR and 0.17% of MDR isolates were *Staphylococcus intermedius* (Table 1).

CoNS group constituted 5.73% of AMR and 3.94% of MDR isolates. Within CoNS, *S. epidermidis* represented the majority of both AMR (87.72%) and MDR (72.00%) isolates, followed by *Staphylococcus* saprophyticus, which contributed 5.26% of AMR and 4.00% of MDR isolates. *Staphylococcus felis* and *Staphylococcus* *lentus* together contributed 1.75% of AMR isolates (Table 1).

The coagulase-variable group accounted for 2.51% of AMR and 3.47% of MDR isolates. Within this group, unspecified species constituted 96.0% of AMR isolates, with 77.27% of these being MDR. *Staphylococcus* *schleiferi* made up 4.00% of AMR and 22.73% of MDR isolates within this group (Table 1).

#### Distribution of AMR across different antimicrobial classes

A total of 16 antimicrobials from 9 different classes were evaluated for susceptibility among the *Staphylococcus* isolates (Table 2). Overall, 39.64% of isolates exhibited resistance to penicillins. Within this class, the highest resistance was observed against penicillin (63.88%), followed by ampicillin (20.78%), while amoxicillin exhibited the least resistance (15.35%). Similarly, among MDR isolates, penicillin resistance was most common (66.95%), followed by resistance to ampicillin (17.57%) and amoxicillin (15.84%) (Table 2).

**Table 2:** Antimicrobial drug resistance categorized by antimicrobial class in canine *Staphylococcus* samples submitted to diagnostic laboratories in South Africa, 2012–2017.

Class	Sub–class	AMR^b^ isolates			MDR^c^ isolates		
	
		*n*	%	95%CI^a^	*n*	%	95%CI^a^
Aminoglycosides		363	22.31		226	13.89	
	Amikacin	108	29.75	29.27–30.23	66	29.20	27.77–30.63
	Gentamicin	176	48.48	48.00–48.96	102	45.13	43.57–46.69
	Kanamycin	79	21.76	21.27–22.25	58	25.66	24.09–27.23
Penicillins		645	39.64		461	28.33	
	Amoxicillin/Clavulanic Acid	99	15.35	14.85–15.85	73	15.84	14.90–16.78
	Penicillin	412	63.88	63.40–64.36	307	66.59	65.37–67.81
	Ampicillin Amoxicillin	134	20.78	20.30 –21.26	81	17.57	16.63–18.51
Cephalosporins		134	8.24		56	3.44	
	Cephalexin	58	43.28	42.78 –43.78	32	0.01	0.59–0.61
	Cefovecin	76	56.72	56.23 –57.21	24	0.00	–0.60–0.60
Sulfonamides							
	Sulfamethoxazole	89	5.47	5.00–5.94	66	4.06	3.30–4.82
Tetracyclines							
	Tetracycline	68	4.18	3.70–4.66	40	2.46	1.80 –3.12
Lincosamides							
	Clindamycin	46	2.83	2.35–3.31	38	2.34	1.68 –3.00
Fluoroquinolones		151	9.28		116	10.56	
	Fluoroquinolones	103	68.21	67.71–68.71	78	46.99	45.43–48.55
	Enrofloxacin	48	31.79	31.30–32.28	38	22.89	21.33–24.45
Macrolides		98	6.02		76	6.92	
	Erythromycin	62	63.27	62.77–63.77	51	67.11	65.66–68.56
	Tilmicosin	36	36.73	36.25–37.21	25	32.89	30.33–35.45
Chloramphenicols							
	Chloramphenicol	33	2.03	1.55–2.51	20	1.23	0.57–1.89

a 95% CI=95% Confidence interval,

b AMR=Antimicrobial resistance,

c MDR=Multidrug resistant

Resistance to aminoglycosides was noted in 22.31% of isolates. Within this class, the highest proportion of resistance was to gentamicin (48.48%), followed by amikacin (29.75%) and kanamycin (21.76%). Among MDR isolates resistant to aminoglycosides, gentamicin resistance was again most prevalent (45.13%), followed by resistance to amikacin (29.20%) and kanamycin (25.13%) (Table 2).

Resistance to cephalosporins was observed in 8.24% of AMR isolates and 3.44% of MDR isolates. Within the cephalosporins, most AMR isolates resistant to the class were resistant to cefovecin (56.72%), while 43.28% were resistant to cefalexin. Notably, none of the MDR isolates were resistant to cefalexin, and only a minimal proportion (0.1%) was resistant to cefovecin (Table 2).

Among sulfonamides, sulfamethoxazole was the only agent tested. Resistance against sulfamethoxazole was found in 5.47% of AMR isolates and 4.06% of MDR isolates (Table 2).

4.8% of AMR isolates were resistant to tetracycline, while only 2.48% of MDR isolates were resistant to tetracycline.

Only clindamycin was tested among lincosamides. Only 2.83% of AMR and 2.34% of MDR isolates demonstrated resistance against clindamycin.

Fluoroquinolone resistance was detected in 9.28% of AMR isolates and 10.56% of MDR isolates. Within this class, most AMR isolates were resistant to fluoroquinolones broadly (68.21%), while 31.29% exhibited resistance specifically to enrofloxacin. Among MDR isolates, 46.99% were resistant to fluoroquino-lones and 22.89% to enrofloxacin (Table 2).

Macrolide resistance was identified in 6.2% of AMR and 6.92% of MDR isolates. Within the macro-lides, erythromycin resistance was most common (63.27% in AMR and 67.11% in MDR isolates), followed by resistance to tilmicosin (36.73% in AMR and 32.89% in MDR isolates).

Resistance to chloramphenicol, the only agent assessed in its class, was noted in 2.03% of AMR isolates and 1.23% of MDR isolates.

Just over half (53.4%) of isolates exhibited resistance to one or two antimicrobial classes, while up to 14.2% demonstrated resistance to six classes. Among MDR isolates, 72.0% exhibited resistance to seven antimicrobials (amoxicillin-clavulanic acid, penicillin, amikacin, gentamicin, erythromycin, tilmicosin, and tetracycline), belonging to four antimicrobial classes: Beta-lactams, aminoglycosides, macrolides, and tetracyclines.

### Temporal patterns of AMR and MDR

**Figure 1:**
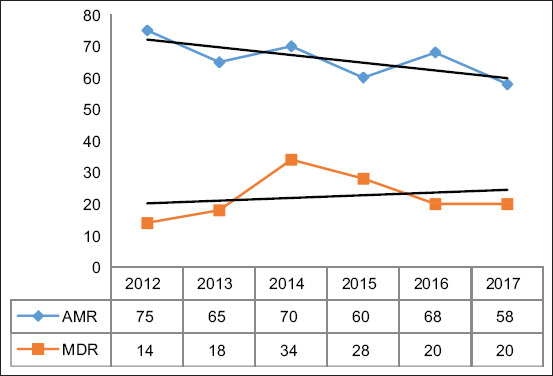
Annual temporal trend of antimicrobial resistance and MDR *Staphylococcus* species from canine clinical specimens submitted to a veterinary diagnostic laboratory, South Africa, 2012–2017.

The proportion of AMR isolates fluctuated throughout the study period. However, the fitted trend line (Figure 1) indicated an overall decreasing trend in AMR over the study period. Conversely, although the proportion of MDR isolates also varied, the fitted trend line showed an increasing trend in MDR during the same period (Figure 1).

### Predictors of antimicrobial and MDR

Variables found to be significantly associated with AMR or MDR at a liberal p-value threshold of ≤0.2 in univariate models were included in the respective multivariate models.

For AMR, significant predictors in the multi-variate model, included the year of isolation, bac- terial species, and specimen type (Table 3). A significant association (p < 0.001) was observed between species and AMR. *S. pseudintermedius* had significantly higher odds of AMR (AOR = 2.23; 95% CI: 1.72–1.84) compared with *S. epidermidis* (reference category). Conversely, *S. aureus* had significantly lower odds of resistance (AOR = 0.5; 95% CI: 0.4–0.7) compared to the reference group (Table 3).

**Table 3:** Predictors of AMR among *Staphylococcus* species from canine clinical isolates submitted to a veterinary diagnostic laboratory, South Africa, 2012–2017.

Variable	OR^a^	95% CI^b^	p-value
Species of organism			
*Staphylococcus pseudintermedius*	2.23	1.7–2.8	<0.001
*Staphylococcus aureus*	0.7	0.4–0.7	<0.001
*Staphylococcus epidermidis*	-	-	-
Specimen type			
Ear	1.6	0.5–4.3	0.221
Respiratory	0.6	0.3–1.2	0.071
Skin	1.8	1.1–2.4	<0.001
Urinary	0.8	0.5–1.7	0.760
Other	-	-	-
Year			
2012	0.7	0.4–1.7	0.761
2013	0.5	0.2–1.2	0.057
2014	1.4	0.5–4.5	0.321
2015	0.9	0.5–1.6	0.395
2016	1.5	0.6–4.0	0.232
2017	-	-	-

a OR=Odds ratio,

b 95% CI=95% Confidence interval, AMR=Antimicrobial resistance

Specimen type also significantly influenced AMR: Isolates from the skin were almost twice as likely to be AMR (AOR = 1.8; 95% CI: 1.1–2.4) compared with those from other specimens. Isolates from the respiratory system showed marginally lower odds (AOR = 0.6; 95% CI: 0.3–1.2; p = 0.071). However, there was no difference in the odds of resistance between isolates from the ear and urinary system and the reference group (Table 3). Likewise, the year of isolation was not a significant predictor of AMR.

Regarding MDR, only specimen type and age were significantly associated with MDR in univariate analyses, and where thus included in the multivariate analysis (Table 4). In the multivariate model, isolates from skin specimens were 12 times as likely to be MDR (AOR = 12.2; 95% CI: 3.4–58.2) compared to isolates from other specimens. Similarly, isolates from ear specimens were four times as likely to be MDR (AOR = 3.6; 95% CI: 0.5–22.3), although the difference was not statistically significant (Table 4).

**Table 4:** Predictors of MDR among *Staphylococcus* isolates from specimens submitted to a veterinary diagnostic laboratory in South Africa, 2012–2017.

Variable	AOR^a^	95% CI^b^	p-value
Specimen type			
Ear	3.6	0.5–22.3	0.142
Respiratory	2.1	0.6–11.6	0.246
Skin	12.2	3.4–58.2	0.001
Urinary	0.7	0.3–2.58	0.721
Other	-	-	-
Age (years)			
≤2	0.5	0.3–1.1	0.162
3–4	1.4	1.2–2.7	0.032
5–6	1.7	0.5–4.2	0.251
7–8	1.5	0.6–3.4	0.265
>8	-	-	-

a AOR=Adjusted odds ratio,

b 95% CI=95% Confidence interval, MDR=Multidrug resistant

With respect to age, isolates from dogs aged 3–4 years had higher odds of being MDR (AOR = 1.7; 95% CI: 1.2–2.7) compared to the reference group (>8 years). No significant differences were observed for other age groups compared to the reference group (Table 4).

## DISCUSSION

### Distribution of resistance across demographic characteristics and species of organism

The discovery of antimicrobials has been one of the key developments in both human and veterinary medicine over the past century [15]. However, due to a combination of factors, including an increase in the therapeutic use of antimicrobials for both humans and domestic animals, AMR has become a scientific and public health concern globally [13].

Resistance to commonly used antimicrobials, as observed in this study, particularly acquired MDR among CoPS is a growing concern in both human and veterinary medicine [15]. Antimicrobial drug resistance, MDR, and methicillin resistance among canine *Staphylococcus* isolates, coupled with the public health implications of zoonotic transmission of these organisms, highlight the importance of monitoring antimicrobial susceptibility patterns [14].

Most AMR (52.46%) and MDR (57.73%) *Staphylococcus* isolates were from male dogs in the current study, which is likely a result of a combination of behavioral, physiological, and environmental factors. Male dogs typically engage in more outdoor activities and interactions with other animals and environments, increasing their risk of encountering and acquiring resistant bacteria. Outdoor behaviors such as roaming and marking territory expose male dogs to various sources of infection, including contaminated soil, water, and other animals [16]. Nocera *et al*. [17] and Tyson *et al*. [18] have consistently demonstrated that outdoor exposure is a risk factor for developing resistant infections in animals. Studies by Tyson *et al*. [18], Becker *et al*. [19], Hammerum *et al*. [20], and Lee *et al*. [21] have also shown that pets with increased outdoor activities are more susceptible to resistant infections due to their frequent interactions with diverse environments and animals.

The age of dogs played a role in the prevalence of AMR and MDR *Staphylococcus* isolates, as observed in the current study. The study revealed that a higher proportion of both AMR and MDR isolates came from dogs aged 2–4 years compared with other age groups. Specifically, dogs aged 2–4 years contributed most of the AMR and MDR isolates. This observation can be attributed to several factors related to their behavior, physiology, and exposure to environmental reservoirs of resistant bacteria. Dogs aged 2–4 years are typically more active and exploratory. As observed for male dogs, 2–4-year-old dogs may have higher exposure to resistant bacteria due to increased outdoor activities, such as roaming and interactions with other animals. These behaviors can elevate the risk of developing infections and, subsequently, resistant strains. This finding highlights the importance of considering environmental influences and behavioral characteristics when assessing the risk of resistance in companion animals.

Physiological factors also contribute to the increased susceptibility of dogs in the 2–4-year age group to resistant infections. This age group corresponds to young adulthood in dogs, during which their immune systems are maturing but may not yet be fully developed or optimized to combat infections effectively [22]. Immune system maturation affects the ability of cells to respond to bacterial challenges, potentially leading to prolonged or recurrent infections that favor the development of resistance.

Conversely, younger dogs under 2 years of age exhibited the lowest proportions of both AMR and MDR isolates. This lower prevalence can be attributed to the limited exposure to antibiotics and infectious agents. Puppies typically receive veterinary care but may not encounter as many opportunities for exposure to antibiotics during their early life stages. In addition, the immune systems of these young animals are still developing, potentially resulting in more effective responses to infections and reducing the likelihood of persistent colonization with resistant bacteria [23].

The majority of both AMR and MDR isolates were CoPS isolates, highlighting their role in resistance development. *S. pseudintermedius* emerged as the predominant CoPS species, contributing a substantial proportion of both AMR and MDR isolates. This species is an opportunistic pathogen in dogs, commonly associated with skin and wound infections [17]. The prevalence of resistant infections underscores its adaptive capability and the challenges posed by clinical management and infection control.

Tyson *et al*. [18] have documented the genetic basis of resistance in *S. pseudintermedius*, including genes encoding resistance to beta-lactams, fluoroquinolones, and other antimicrobial agents. This genetic resilience contributes to its high prevalence in both community and hospital settings, necessitating tailored therapeutic approaches and stringent infection control measures.

Although less prevalent overall, CoNS also contributed to the number of resistant isolates observed in this study. *S. epidermidis*, the most common species among CoNS, is associated with high resistance levels, particularly in healthcare-associated infections involving indwelling medical devices [19]. The ability of a host to form biofilms contributes to persistent infections and challenges in the eradication of resistant strains.

Although less common, *S. saprophyticus* displayed resistance profiles indicative of its adaptation to environmental niches and potential transmission routes in veterinary settings. The presence of CoNS species such as *S. felis* and *S. lentus* further underscores the diversity of resistant *Staphylococci* circulating among canine populations, necessitating surveillance to monitor emerging resistance mechanisms [20].

Although less prevalent, the coagulase-variable group showed interesting findings regarding resistance. The predominance of unspecified species within this group highlights gaps in the current understanding and surveillance of resistance dynamics. *S. schleiferi*, a notable member, demonstrated significant resistance, particularly among MDR isolates, indicating its potential as an emerging pathogen in veterinary medicine [21].

### Temporal patterns of AMR and MDR

The temporal trends of AMR and MDR among *Staphylococcus* isolates over the study period (2012–2017) fluctuated. Several studies have reported similar trends, reflecting the dynamic nature of bacterial resistance over time. For instance, a study by Hamzah *et al*. [24] documented fluctuating trends in AMR and MDR among *Staphylococcus* species over a 5-year period, similar to the trends observed in our present study. Similarly, Zulkeflle *et al*. [25] reported varying patterns of AMR and MDR in *Staphylococcus* isolates, suggesting the complexity of resistance mechanisms.

In line with the observed fluctuations, a study by Aijaz *et al*. [26] highlighted the dynamic nature of AMR and MDR, emphasizing the need for continuous surveillance and data-driven intervention strategies. In addition, Munita and Arias [27] found that although AMR rates may exhibit more variability, MDR rates tend to show a more consistent pattern over time, echoing the trends observed in our study.

The differences in the trends of AMR and MDR, as noted in the present study, have also been documented by Scaglione *et al*. [28], who attributed the varying trends to differences in the mechanisms or drivers of resistance to single antimicrobial agents versus multiple agents. Furthermore, the effectiveness of interventions targeting MDR compared with those targeting resistance against single antimicrobial agents has been discussed in studies by Murugaiyan *et al*. [29] and Bojang *et al*. [30].

Hawkey [31] and Guilhelmelli *et al*. [32] have highlighted the challenges in fully controlling resistant bacterial strains, particularly those resistant to multiple antimicrobial classes. This study’s findings underscore the ongoing need for comprehensive strategies to combat AMR and MDR in *Staphylococcus* isolates.

### Antimicrobial drug resistance observed against the different antimicrobial classes

The data demonstrated varying levels of resistance across different classes of antimicrobials. Notably, the highest level of AMR was observed against common- ly used antimicrobials such as penicillins, followed by aminoglycosides, fluoroquinolones, and cephalo-sporins. This widespread resistance, particularly against commonly used classes like betalactams, was expected because of the high selection pressure associated with frequent usage of these classes of antimicrobials [33].

Within the beta-lactam class, the highest resistance was observed against penicillin. This underscores the need to understand antibiotic resistance within specific classes to tailor treatment approaches and develop targeted interventions [34].

The present study also revealed variations in MDR patterns, with the highest rate of involvement in MDR observed against beta-lactams. Penicillin was again the most involved agent within this category. MDR poses a significant challenge in clinical settings because it limits treatment options and increases the risk of treatment failure.

Variations in resistance rates were observed within specific subclasses. For example, among aminoglycosides, gentamicin showed the highest resistance compared with other members of the class. Understanding these nuances is essential for informed antibiotic selection and treatment planning [35].

It is also worth noting that certain subclasses exhibited low levels of resistance, such as cefalexin and cefovecin. While this observation is promising, it underscores the importance of preserving the effectiveness of existing antimicrobials within classes through prudent use and surveillance.

### Predictors of AMR and MDR

This study provided insights into the factors asso-ciated with AMR and MDR in *Staphylococcus* infections in dogs. Key variables such as year of isolation, age, species of organism, and specimen type were analyzed to determine their influence on resistance patterns.

The species of the organism was significantly associated with AMR, highlighting the need to consider specific staphylococcal species when addressing resistance issues. For example, *S. pseudintermedius* exhibited higher odds of AMR compared with *S. epidermidis*, as reflected by an AOR of 2.23. This finding is consistent with previous research by Prior *et al*. [36], which identified *S. pseudintermedius* as a prominent pathogen in dogs, frequently associated with skin, otitis, and wound infections, and also known for its high resistance rates [36].

The presence of the *mecA* gene that confers methicillin resistance in these organisms has been well documented [37]. In contrast, *S. aureus* was associated with significantly lower odds of being resistant compared to *S. epidermidis*. This is an intriguing result considering the virulence of methicillin-resistant *S. aureus* in both human and veterinary medicine [38]. The lower odds of resistance noted among *S. aureus* in this study might be due to the specific population dynamics or variations in antimicrobial use practices in the studied canine population.

The type of specimen from which the isolates were obtained was another significant predictor of resistance. Skin isolates were twice as likely to be AMR compared with the reference group (other specimen types). This is consistent with the understanding that skin infections, often treated with various antibiotics, are hotspots for resistance development due to high selective pressure they are subjected to [39].

The higher odds of resistance noted among isolates from the ear, although not statistically significant, suggest that otitis externa may similarly be a critical site for resistant infections, likely due to recurrent and prolonged antibiotic treatments [40]. Conversely, although not significant, the lower odds of isolates from the respiratory and urinary systems being resistant may reflect differences in the antibiotic treatment regimens commonly employed for these types of infections or differences in pathogen expo-sure [41].

Dogs aged 3–4 years had higher odds of being MDR compared to isolates from older dogs (>9 years), suggesting that younger adult dogs are more prone to harboring MDR *Staphylococci* [42]. This could be attributed to their higher activity levels and increased likelihood of exposure to various environments and other animals, thereby enhancing their risk of acquiring and spreading resistant bacteria [43].

Interestingly, the year of isolation was not a significant predictor of AMR, indicating that resistance patterns remained relatively stable over the study period [44]. This stability suggests that the driving factors for resistance in this population are more related to specific practices and exposures than to temporal trends [45, 46].

## CONCLUSION

This study revealed a high prevalence of AMR (61.20%) and MDR (39.00%) among *Staphylococcus* spp. isolated from canine clinical specimens in South Africa. *S. pseudintermedius* emerged as the predominant species associated with resistance, particularly against beta-lactams, aminoglycosides, and fluoroquinolones. Higher proportions of AMR and MDR isolates were observed in male dogs and those aged 2–4 years, likely due to behavioral and physiological factors increasing exposure risk. Skin specimens were significantly associated with higher odds of both AMR and MDR, underscoring the need for targeted management of dermatological infections.

The findings have important practical implications for veterinary practice and public health. They highlight the urgent necessity for antimicrobial stewardship programs tailored to companion animals, the refinement of empirical treatment protocols based on local resistance data, and heightened surveillance to monitor emerging resistance trends and mitigate zoonotic transmission risks.

A key strength of the study lies in its large sample size, spanning 6 years, and the use of standardized antimicrobial susceptibility testing, providing critical baseline epidemiological data for canine *Staphylococcus* infections in the region. However, limitations include the retrospective nature of the study, potential bias due to the absence of clinical outcome data, and a lack of molecular characterization of resistance mechanisms such as the presence of the *mecA* gene.

Future research should focus on prospective studies integrating molecular diagnostics to detect specific resistance genes, longitudinal monitoring to assess evolving resistance patterns, and the development of predictive models to inform clinical decision-making. In addition, investigating resistance dynamics across different regions and veterinary settings would provide a broader understanding of AMR dissemination in companion animal populations.

The high burden of AMR and MDR among canine *Staphylococcus* isolates in this study underscores the critical need for continuous surveillance, judicious antimicrobial use, and integrated One Health strategies to safeguard both animal and human health [47].

## DATA AVAILABILITY

The data supporting the findings of this study are available upon reasonable request and subject to specific conditions. For inquiries regarding data access, including requests for collaboration or data sharing agreements, please contact M. Henton, Bacteriologist, at henton@vetdx.co.za. Requests will be evaluated on a case-by-case basis, considering the request, compliance with relevant regulations, and any associated agreements or protocols.

## AUTHORS’ CONTRIBUTIONS

TTS, JWO, and DNQ: Conceptualized the study. TTS: Acquired and curated data, conducted the formal analysis, and drafted the manuscript. JWO and DNQ: Supervised the study and reviewed and edited the manuscript. All authors have read and approved the final version of the manuscript for publication.
